# A Pharmacovigilance Study in First Episode of Psychosis: Psychopharmacological Interventions and Safety Profiles in the PEPs Project

**DOI:** 10.1093/ijnp/pyv121

**Published:** 2015-10-27

**Authors:** Miquel Bioque, Adrián Llerena, Bibiana Cabrera, Gisela Mezquida, Antonio Lobo, Ana González-Pinto, Covadonga M. Díaz-Caneja, Iluminada Corripio, Eduardo J. Aguilar, Antoni Bulbena, Josefina Castro-Fornieles, Eduard Vieta, Amàlia Lafuente, Sergi Mas, Mara Parellada, Jerónimo Saiz-Ruiz, Manuel J. Cuesta, Miguel Bernardo, PEPs GROUP

**Affiliations:** Barcelona Clínic Schizophrenia Unit, Hospital Clínic de Barcelona, CIBERSAM, Barcelona, Spain (Drs Bioque, Cabrera, Mezquida, and Bernardo); CICAB-CAIBER Centro de Investigación Clínica, Hospital Universitario, Servicio Extremeño de Salud SES, Facultad de Medicina Universidad de Extremadura, CIBERSAM, Badajoz, Spain (Dr Llerena); Instituto de Investigación Sanitaria Aragón (IIS Aragón), University of Zaragoza, Zaragoza, Spain (Dr Lobo); Department of Psychiatry, Hospital Universitario de Alava, CIBERSAM, University of the Basque Country, Vitoria, Spain (Dr González-Pinto); Child and Adolescent Psychiatry Department, Hospital General Universitario Gregorio Marañón, IiSGM, CIBERSAM, School of Medicine, Universidad Complutense, Madrid, Spain (Drs Díaz-Caneja and Parellada); Department of Psychiatry, Hospital de Sant Pau, CIBERSAM, Barcelona, Spain (Dr Corripio); Clinic Hospital Valencia, INCLIVA, CIBERSAM, Valencia University, Valencia, Spain (Dr Aguilar); Department of Psychiatry, Hospital del Mar, Barcelona, IMIM (Hospital del Mar Medical Research Institute), Barcelona, Spain (Dr Bulbena); Department of Child and Adolescent Psychiatry and Psychology, SGR-489, Neurosciences Institute, Hospital Clínic of Barcelona, IDIBAPS, CIBERSAM, University of Barcelona, Spain (Dr Castro-Fornieles); Hospital Clínic de Barcelona, Universitat de Barcelona, IDIBAPS, CIBERSAM, Barcelona, Spain (Dr Vieta); Department of Pathological Anatomy, Pharmacology and Microbiology, University of Barcelona, Institut d’Investigacions Biomèdiques August Pi i Sunyer (IDIBAPS), CIBERSAM, Barcelona, Spain (Drs Lafuente and Mas); Hospital Ramon y Cajal, Universidad de Alcala, IRYCIS, CIBERSAM, Madrid, Spain (Dr Saiz-Ruiz); Servicio de Psiquiatría, Complejo Hospitalario de Navarra, Instituto de Investigación Sanitaria de Navarra, Pamplona, Spain (Dr Cuesta); Universitat de Barcelona, IDIBAPS, Barcelona, Spain (Dr Bernardo).

**Keywords:** Antipsychotic, first episode psychosis, pharmacovigilance, polytherapy, psychotropic drugs, schizophrenia.

## Abstract

**Background:**

The characterization of the first episode of psychosis and how it should be treated are principal issues in actual research. Realistic, naturalistic studies are necessary to represent the entire population of first episode of psychosis attended in daily practice.

**Methods:**

Sixteen participating centers from the PEPs project recruited 335 first episode of psychosis patients, aged 7 to 35 years. This article describes and discusses the psychopharmacological interventions and safety profiles at baseline and during a 60-day pharmacovigilance period.

**Results:**

The majority of first episode of psychosis patients received a second-generation antipsychotic (96.3%), orally (95%), and in adjusted doses according to the product specifications (87.2%). A total of 24% were receiving an antipsychotic polytherapy pattern at baseline, frequently associated with lower or higher doses of antipsychotics than the recommended ones. Eight patients were taking clozapine, all in monotherapy. Males received higher doses of antipsychotic (*P*=.043). A total of 5.2% of the patients were being treated with long-acting injectable antipsychotics; 12.2% of the patients received anticholinergic drugs, 12.2% antidepressants, and 13.7% mood stabilizers, while almost 40% received benzodiazepines; and 35.52% reported at least one adverse drug reaction during the pharmacovigilance period, more frequently associated with higher antipsychotic doses and antipsychotic polytherapy (85.2% vs 45.5%, *P*<.001).

**Conclusions:**

These data indicate that the overall pharmacologic prescription for treating a first episode of psychosis in Spain follows the clinical practice guideline recommendations, and, together with security issues, support future research of determinate pharmacological strategies for the treatment of early phases of psychosis, such as the role of clozapine, long-acting injectable antipsychotics, antipsychotic combination, and the use of benzodiazepines.

## Introduction

A first episode of psychosis (FEP) is a frequent condition suffered by approximately 3% of the general population, beginning usually in youth (Perala et al., 2007). The characterization of the population with a FEP has become an area of main interest for research (Bernardo and Bioque, 2014), with a growing number of studies in the last decade worldwide (McEvoy et al., 2007; Bertelsen et al., 2008; Castro-Fornieles et al., 2008; Kahn et al., 2008; Bertani et al., 2011). The conduct of longitudinal research in early phases of the illness has become especially relevant, because it avoids the effect of confounding variables such as the influence of antipsychotic treatment, comorbidities, or chronicity (Kahn et al., 2008).

Antipsychotic medications are known to improve the outcomes and reduce the relapse rates in early phases of schizophrenia and related disorders (Alvarez-Jimenez et al., 2009; Barnes and Psychopharmacology, 2011). However, there is still some controversy about important aspects of the pharmacological treatment of the FEP, including the supposed superior effectiveness of the second-generation antipsychotic (SGA) over the first generation (FGA) (Crossley et al., 2010; Leucht et al., 2013). Currently, the major part of the guidelines on the treatment of schizophrenia focuses on side effects rather than differential efficacy and recommend starting with low doses in FEP, with careful side effect monitoring (American Psychiatric Association, 2004; Deutsche Gesellschaft für Psychiatrie Psychotherapie und Nervenheilkunde, 2006; Grupo de trabajo de la Guía de Práctica Clínica sobre la Esquizofrenia y el Trastorno Psicótico Incipiente. Fòrum de Salut Mental, 2009; National Institute of Clinical Excellence, 2009; Barnes and Psychopharmacology, 2011).

As all pharmacological treatments, all antipsychotics can cause adverse drug reactions (ADRs) (Leucht et al., 2013), but an adequate antipsychotic dosage is related to a decrease in mortality (Torniainen et al., 2014). Some of these side effects may cause symptoms similar to those of schizophrenia, such as secondary negative symptoms (Bernardo et al., 2013), while some subjects may develop side effects that are new symptoms, such as galactorrhea, disrupted sexual function, or sedation. The subjective distress and the functional impairment that result from these ADRs are associated with the poor adherence rates to these treatments (Sendt et al., 2015).

Apart from effectiveness and tolerability, other important issues to elucidate are the role of long-acting injectable antipsychotics (LAIAs) in FEP patients (Patel et al., 2010; Zhornitsky and Stip, 2012; Manchanda et al., 2013; Correll, 2014; Kishimoto et al., 2014; Subotnik et al., 2015), the real rates of antipsychotic polytherapy, and the coadjuvant treatments such as antidepressants or benzodiazepines (Gallego et al., 2012).

In this context, the “Phenotype-genotype and environmental interaction. Application of a predictive model in first psychotic episodes” (PEPs project) was a multicenter, prospective, longitudinal, naturalistic study designed to evaluate clinical, neuropsychological, neuroimaging, biochemical, and genetic variables in a sample of 335 FEP patients in Spain matched with 253 healthy controls (Bernardo et al., 2013). Due to the naturalistic design of the PEPs project, participants maintained their usual treatment. This feature has allowed, for the first time to our knowledge, to get a global picture of the usual treatment and outcomes in patients with a FEP in Spain (Mas et al., 2012), being a unique opportunity to study treatment prescription patterns in a real life sample of 335 FEP subjects, together with the presence of ADRs.

One of the aims of the PEPs Project was to get as close as possible to the actual daily practice with FEP, following a naturalistic design that represented the whole natural history of psychotic disorders (Bernardo et al., 2013). Therefore, one of the strengths of this study was that the age of inclusion was wide, including patients from 7 to 35 years, with the aim of covering the whole range of ages at which a FEP may appear. Previous studies with FEP (especially clinical trials) generally exclude the population with early onset psychosis, which may be a bias because of the worse prognosis of this subset of patients (Arango et al., 2012). This wide window of age pointed to the fact that the average age of this sample tends to be inferior to other large studies with large FEP cohorts such as the OPUS or EUFEST trials (Bertelsen et al., 2008; Kahn et al., 2008). Besides, patients with suicide ideation or a drug use disorder, frequently excluded from FEPs studies, were allowed to participate (Bernardo et al., 2013).

This article aims to describe the prescription patterns in a multicenter, naturalistic study with more than 300 FEP with a wide age range (7–35 years), putting a particular emphasis on practices that are not usually first-line recommendations in international guidelines, such as polypharmacy, the use of LAIAs, or clozapine, and how these patterns were related to security/tolerance issues.

## Methods

### Subjects

The 16 centers participating in the PEPs project recruited a total of 335 patients with a FEP from April 2009 to April 2012. Patients were recruited from 16 centers located throughout the Spanish territory with experience in performing and assessing diagnoses, in the use of semi-structured interviews and clinical scales, and in treating this population. Fourteen of these teams were members of the Centro de Investigaciones Biomédicas en Red en Salud Mental (CIBERSAM), the Spanish network of translational research in neuroscience aspects related to health and mental illness, together with 2 collaborator centers (Bernardo et al., 2013). Every patient who met the inclusion criteria and was attended at these facilities during the recruitment period was invited to participate in the study on either an inpatient or outpatient basis.

The inclusion criteria for patients were: age between 7 and 35 years, presence of first psychotic symptoms (positive symptoms or disorganization) of at least 1 week duration in the last 12 months, and speak Spanish correctly. The exclusion criteria for patients were: (1) mental retardation according to the Diagnostic and Statistical Manual of mental disorders, 4th edition (DSM-IV) criteria (American Psychiatric Association (Washington), 1994), (2) history of head trauma with loss of consciousness, and (3) presence of an organic disease with mental repercussions.

Since this was a naturalistic study, there were no specific guidelines for treatments (drugs and/or psychotherapy). Treatment with antipsychotics did not exceed 12 months at study entry. All scales included in the PEPs project protocol, except those self-administered, were administered by expert clinicians. The rationale for these criteria and the complete clinical protocol used in the PEPs project were previously published elsewhere (Bernardo et al., 2013).

The study was approved by the investigation ethics committees of all participating clinical centers. Informed consent was obtained from all participants. In case of children under 18 years of age, patients assented to participate and parents or legal guardians gave written informed consent before their inclusion.

### Diagnostic, Demographic, and Clinical Data Collection

The Spanish translation of the Kiddie-Sads-Present and Lifetime Version (K-SADS-PL) (Kaufman et al., 1997) was used to assess current and past psychopathology in children and adolescents according to DSM-IV criteria, and the Structured Clinical Interview for DSM Disorders (SCID) parts I and II (SCID-I & II), with a Spanish translation available, was used for adults (American Psychiatric Association, 1994; First et al., 1994, 1999). The psychopathological assessment was performed using the Positive and Negative Syndrome Scale (Kay et al., 1987), the Young Mania Rating Scale (Young et al., 1978), and the Montgomery-Asberg Depression Rating Scale (Montgomery and Asberg, 1979). The Global Assessment of Functioning Scale, and the Children’s Global Assessment Scale were used to measure the global severity of symptoms and level of functioning (Endicott et al., 1976; Shaffer et al., 1983). At baseline, a complete personal and family history was compiled, including a register of lifetime drug history. In every evaluation, the information of which drugs the subjects were taking, the dosage, and the presence of ADRs was collected.

### Calculation of Prescribed Daily Doses

For the first 60 days after the inclusion of the patient in the PEPs project, all psychotropic drugs prescribed to every patient were recorded, independently of the dose and separating different formulations of the same substance. Thus, a number expressing the sum of concomitant prescriptions for each treatment day was obtained.

The prescribed daily dose (PDD) for a drug was defined as the daily dose of a drug formulation, oral or injectable, calculated separately for each treatment day of an individual patient who was treated with this particular drug formulation for at least 3 consecutive days (irrespective of the dose). Different formulations of the same drug were separated. The PDD of LAIAs was calculated by dividing the given dose by the number of days until the next depot injection.

To compare the different antipsychotics between them, the PDDs doses of antipsychotics were converted to an estimated equivalent amount of chlorpromazine (CPZ) following the international consensus (Gardner et al., 2010).

Baseline polypharmacy was registered considering simultaneous treatment in the same patient with one antipsychotic together with an antidepressant, an anticholinergic drug, a mood stabilizer, a benzodiazepine, or another antipsychotic used at the same time.

### ADR Evaluation

To assess in detail the ADRs, 2 procedures were followed: (1) spontaneous reports of ADR; (2) systematic assessment of the effects targeted (like metabolic syndrome, cardiotoxicity or extrapyramidal symptoms) from physical examination (electrocardiogram, antipsychotic plasmatic levels, and general blood tests) and 2 scales administrated in every follow-up visit: (1) the Scale of the Udvalg for Kiniske Undersogelser (Lingjaerde et al., 1987), a comprehensive rating scale designed to assess general side effects of psychotropic drugs; and (2) the Simpson-Angus Scale (Simpson and Angus, 1970), included to evaluate the extrapyramidal side effects. Drug-induced Parkinsonism is a common and poorly tolerated adverse effect, classically related to typical antipsychotics and determinate atypical antipsychotics at higher doses.

Investigators also reported any specific treatment or change in the prescription due to ADR appearance, including antipsychotic discontinuation, dose reduction, or start of an anticholinergic drug.

Those ADRs that made the clinician change the usual practice by, for example, requesting a blood test that was not previously scheduled, sending the patient to the emergency room, or admitting the patient to the hospital were considered as serious.

### Data Processing and Statistical Analysis

The tool GRIDSAM was used for data entry, allowing the different centers to capture the data by means of a multi-grid computerized system, which not only integrates all the available information but also facilitates a more efficient data exploitation and management (Bernardo et al., 2013).

Differences in baseline demographic and clinical characteristics between patients and controls were assessed using Chi-square, *t* test, or nonparametric Mann-Whitney U test, according to the distribution and scales of the variables. Binary logistic regression was performed to assess the impact of different covariates on the likelihood that any ADRs would be reported. A value of *P*<.05 was taken to be statistically significant. Data was managed and analyzed with the IBM SPSS Statistics v.20 (IBM Corp, 2011).

## Results

### Baseline Clinical Characteristics and Psychotropic Drug Treatment

A total of 335 patients with a FEP were included in the PEPs project and completed the baseline visit. Baseline demographic, clinical, and psychotropic drug treatment characteristics are presented in Table 1.

**Table 1.** Baseline Demographic, Clinical, and Psychotropic Drug Treatment Characteristics

	Patients (n = 335)
Age (y)	23.58±6.00
Gender, n (%)	
Male	225 (67.2)
Female	110 (32.8)
Ethnic group, n (%)	
Caucasian	284 (84.8)
Gipsy	6 (1.8)
Maghrebian	8 (2.4)
Sub-Saharan	4 (1.2)
Asian	4 (1.2)
Caribbean	8 (2.4)
Hispanic	17 (5.1)
Other	4 (1.2)
Diagnosis, n (%)	
Affective psychosis	55 (16.4)
Non-affective psychosis	280 (83.6)
Psychopathology scales scores	
PANSS	74.95±24.57
Young	9.26±10.48
Montgomery-Asberg	12.94±9.82
Overall functioning score (GAF/C-GAS)	50.92±19.66
Subjects antipsychotic treatment, n (%)	
No antipsychotics	31 (9.3)
Antipsychotic monotherapy	232 (69.2)
Antipsychotic polytherapy	73 (24)
2 antipsychotics	67 (20)
3 antipsychotics	5 (1.5)
4 antipsychotics	1 (0.3)
Antipsychotic dosage, n (%)	
Lower	12 (3.9)
Adequate	265 (87.2)
Higher	27 (8.9)
Route of antipsychotic, n (%)	
Only oral	291 (94.8)
Only long acting injection	1 (0.3)
Both	15 (4.9)
Subjects with other treatments, n (%)	
Anticholinergics	41 (12.2)
Antidepressants	41 (12.2)
Mood stabilizers	46 (13.7)
Benzodiazepines	130 (38.8)

Affective psychosis includes DSM-IV unipolar depression or bipolar disorder with psychotic features and schizoaffective disorder diagnosis.

Recommended dose range according to the patient age and the specifications of the product to Spain

A total of 280 (83.6%) patients were diagnosed of nonaffective psychotic disorders, and 55 subjects (16.4%) diagnosed with DSM-IV affective disorders (unipolar depression or bipolar disorder) with psychotic features or schizoaffective disorder were classified as “affective psychosis.”

At baseline, patients had been treated with antipsychotics for a mean of 54.08 days. Thirty (9%) patients were not taking any antipsychotic at baseline, including those who were antipsychotic naïve and those who have discontinued previous treatments. Only 3.7% of the prescriptions included a FGA. Of those subjects who were taking antipsychotics at baseline, 73 (24%) were receiving an antipsychotic polytherapy pattern (detailed antipsychotic mono/polytherapy patterns in supplementary Data 1). The rates of antipsychotic polytherapy in the nonaffective vs affective diagnosis groups were almost identical. The most frequent patterns of prescription were monotherapy of olanzapine (n=76), risperidone (n=74), and aripiprazole (n=32). The most frequent antipsychotic polytherapy patterns were risperidone plus olanzapine (n=15) and aripiprazole plus olanzapine (n=9). Eight patients with a FEP were taking clozapine, all of them in monotherapy and all diagnosed with a nonaffective psychotic disorder.

A total of 87.2% of the patients were receiving adequate doses of antipsychotic, according to the recommended dose range in the specifications of the product for Spain for the patient age (AEMPS, 2014) (see online supplementary Data 2). Patients with polytherapy received more frequently lower doses (8.2% vs 2%, *P*=.028) or higher (16.4% vs 6.5%, *P*=.014) of antipsychotics than the recommended ones compared with patients receiving monotherapy, with a moderate effect size (Cramer’s V= 0.2, *P*=.002).

Sixteen (5.2%) of the FEP patients included in the PEPs project were being treated with LAIAs. All these treatments were risperidone LAI, and all except one went with oral supplementation.

Patients diagnosed of affective psychoses received antidepressant drugs (21.8% vs 10.4%, *P*=.02) and mood stabilizers (45.5% vs 7.5, *P*<.001) more frequently than nonaffective psychoses.

### Drug Treatment during the 2-Month Pharmacovigilance Period

Patients were followed in a pharmacovigilance period during the first 60 days after their inclusion in the PEPs project. Baseline treatment was continued during this period. Any changes or discontinuations were registered. Seventeen patients (4.78%) were not taking any antipsychotic during this period, and 14 patients (4.1%) did not complete the follow-up study, so 302 FEP subjects completed the 2-month follow-up study with at least one antipsychotic prescription.

During this period, a total of 420 antipsychotic prescriptions were made, 1.38 per patient being treated. Table 2 presents the PDDs of antipsychotic medications during the pharmacovigilance period.

**Table 2.** Prescribed Daily Dose (PDD) of Antipsychotic Medications during the 2-Month Pharmacovigilance Period

Antipsychotic	Number of Patients (%)	PDD Mean Dose (mg)	PDD Range	Chlorpromazine Equivalent Mean Dose
Risperidone	123 (29.3)	3.79 (3.36–4.21)	0.5–12	379.34 (336.74–421.95)
Olanzapine	114 (27.1)	12.51 (11–14.02)	2.5–40	375.55 (330.22–420.87)
Aripiprazole	63 (15)	13.37 (11.27–15.47)	5–30	267.54 (225.5–309.59)
Paliperidone	36 (8.6)	6.66 (5.27–8.05)	3–18	444.48 (352.01–536.94)
Quetiapine	35 (8.3)	311.35 (240.51–382.19)	50–900	249.08 (192.41–305.75)
Amisulpride	16 (3.8)	580.20 (330.05–830.35)	50–1600	498.97 (283.84–714.1)
Clozapine	14 (3.3)	162.85 (97.54–228.17)	25–400	244.28 (146.31–342.25)
Haloperidol	9 (2.1)	6.54 (2.89–10.19)	2.5–15	392.66 (173.91–611.41)
Ziprasidone	3 (0.7)	62.44 (-61.0433–185.93)	60–160	234.16 (-228.91–697.23)
Perfenazine	3 (0.7)	8	8	160
Clotiapine	2 (0.5)	20	20	120
Zuclopenthixol	1 (0.2)	100	100	1200
Pimozide	1 (0.2)	1.5	1.5	112.5

PPD is shown in milligrams; the 95% confidence intervals of the mean are given in brackets.

Drugs prescribed at the highest equivalent doses of CPZ were paliperidone, amisulpride, and zuclopenthixol, with higher doses than those collected in the ATC/DDD index of the World Health Organization (WHO, 2014). Quetiapine, ziprasidone, and clozapine were prescribed in the lowest equivalent doses.

There was a statistically significant decrease in the antipsychotic dosage between baseline visit and the 2-month visit (t=5.18, *P*<.001) but not in the number of subjects reporting any ADRs (81 vs 73).

Table 3 presents the cumulative daily dose of antipsychotics, separated by different groups. Males received higher doses of antipsychotics than women (*P*=.043). The patients receiving anticholinergic drugs were also those with higher doses of antipsychotic.

**Table 3.** Cumulative Daily Dose of Antipsychotics, Separated by Gender, Diagnosis, and Concomitant Treatment with Anticholinergic Drugs, Antidepressants, Mood Stabilizers, and Benzodiazepines

			Statistic	*P*-Value
Gender	Males (n=203)	Females (n=99)		
	529.99±26.93	414.9±23.78	U=11487.5	.043
Diagnosis	Nonaffective psychosis (n=253)	Affective psychosis (n=49)		
	494.12±20.91	482.67±59.14	t=0.21	.83
Use of anticholinergic drugs	Yes (n=39)	No (n=263)		
	705.34±63.25	460±20.21	t=4.23	<.001
Use of antidepressants	Yes (n=39)	No (n=263)		
	476.08±63.91	494±20.88	t=-0.31	.75
Use of mood stabilizers	Yes (n=40)	No (n=262)		
	528.94±69.77	486.66±20.4	t=0.71	.473
Use of benzodiazepines	Yes (n=118)	No (n=184)		
	524.98±32.58	471.28±25.12	t=1.31	.18

The mean dose is expressed as chlorpromazine equivalents (mg/d).

### ADRs during the Pharmacovigilance Period

A total of 119 patients (35.52%) reported at least one ADR during the pharmacovigilance period. A total of 181 ADRs were reported (collected in Table 4), none of which was reported as serious.

**Table 4.** Adverse Drug Reactions (ADRs) Reported during the Pharmacovigilance Period

	Number of Patients (% Total Patients; n=335)
Number of ADRs reported	
None	216 (64.48)
1	74 (22.08)
2	30 (8.95)
3	13 (3.88)
4	2 (0.6)
Types of ADRs	
Extrapyramidal symptoms	58 (9.9)
Sedation	44 (7.5)
Headache	4 (0.7)
Weight gain	19 (3.2)
Sexual side effects	11 (1.9)
Cognitive/negative	9 (1.5)
Sialorrea	3 (0.5)
Hormonal	12 (2)
Others	21 (3.6)
Treatment for ADRs	
Antipsychotic dose reduction	13 (3.8)
Antipsychotic change	15 (4.5)
Anticholinergic	29 (8.6)
Others	9 (2.6)

The appearance of ADRs was related to higher doses of antipsychotic prescriptions; the mean daily CPZ dose of the group of patients who reported at least one ADR was higher (598.9 vs 482.22mg; *t* test=2.32, *P*=.021).

The percentage of patients who reported at least one ADR during the pharmacovigilance period was higher in those patients treated with antipsychotic polytherapy than in those treated with monotherapy (85.2% vs 45.5%), showing statistical significant differences (χ^2^=15.1; *P*<.001).

Direct logistic regression was performed to assess the impact of these 2 factors (antipsychotic doses and antipsychotic polytherapy) on the likelihood that any ADRs would be reported. The full model containing all predictors was statistically significant (χ^2^ [2, n=237]=18.76, *P*<.001), indicating that the model was able to distinguish between patients who did and did not report ADRs. The model as a whole explained between 7.6% (Cox and Snell R square) and 10.1% (Nagelkerke R squared) of the variance in ADR presence and correctly classified 61.6% of cases. Only antipsychotic polytherapy made a unique statistically significant contribution to the model with an odds ratio of .206 (*P*=.008), indicating that using a polytherapy treatment increased 4.85 (1/0.206) times the likelihood of reporting having any ADRs, controlling for other factors in the model.

On the other hand, other approaches to find variables associated with reports of ADRs, related to the possible interaction between age, gender, and nonaffective/affective diagnosis, the time of evolution of the disease, or the use of other psychotropic drugs, did not reach a statistically significant relationship.

In those patients treated with monotherapy, the appearance of ADRs was related more to the prescription of quetiapine (62.2% reported at least one ADR) and risperidone (50%), while the least were clozapine (25%) and amisulpride (33%).

Sixty-eight (22.5%) of the 302 cases that completed the pharmacovigilance period reported a specific treatment for their adverse events, including antipsychotic stop, dose reduction, or initiation of an anticholinergic drug (see Table 4 for details).

Table 4 also shows strategies reported by the investigators to alleviate ADRs. An antipsychotic dose reduction was recommended in 13 patients, with a mean dose reduction of 21.1% in CPZ equivalents. In 15 patients, an antipsychotic change was introduced; the change to aripiprazole in monotherapy was the most frequent (n=7).

## Discussion

This article describes the baseline treatment characteristics and a 2-month pharmacovigilance period of the 335 patients with a FEP included in the PEPs project, a multicenter, prospective, longitudinal, naturalistic, follow-up study conducted at 16 research sites in Spain. According to these data, after suffering a FEP, the majority of patients receive a SGA (96.3%), via oral administration (95%), in adjusted dosage according to the product specification in our country (87.2%), and more frequently in monotherapy (68.7%). Together, these data offer an overall picture of the pharmacologic prescription for treating this population in Spain.

One of the main objectives of the present article was ascertain to what extent drug prescriptions followed the recommendations of the principal clinical practice guidelines. At present, there is a clinical practice guideline for schizophrenia and incipient psychotic disorder driven by the Spanish Ministry of Health in which evidence-based recommendations are collected (Grupo de trabajo de la Guía de Práctica Clínica sobre la Esquizofrenia y el Trastorno Psicótico Incipiente. Fòrum de Salut Mental, 2009). Several international guidelines, such as those of the German Society of Psychiatry, Psychotherapy and Nervous Disease (Deutsche Gesellschaft für Psychiatrie Psychotherapie und Nervenheilkunde, 2006), the American Psychiatric Association (American Psychiatric Association, 2004), and the National Institute of Clinical Excellence (National Institute of Clinical Excellence, 2009), publish similar recommendations. Regarding specific pharmacological interventions in FEP, the following core principles are outlined in these guidelines (American Psychiatric Association, 2004; Deutsche Gesellschaft für Psychiatrie Psychotherapie und Nervenheilkunde, 2006; Grupo de trabajo de la Guía de Práctica Clínica sobre la Esquizofrenia y el Trastorno Psicótico Incipiente. Fòrum de Salut Mental, 2009; National Institute of Clinical Excellence, 2009). According to the current state of research on pharmacological intervention in FEP, there is not enough scientific evidence to indicate a FGA vs a SGA. However, some studies indicate that there could be greater adherence with SGAs, supporting their use as a first election (Zhang et al., 2013). The onset of the administration of SGA antipsychotics should be at low doses. If there is no response to treatment, switch to another SGA and evaluate the result for 6 to 8 weeks. If while using an SGA an adverse effect occurs, one might consider switching to a FGA. It is recommended to use clozapine in cases where there is no response to treatment, low adherence, or persistent risk of suicide.

In 2009 the PORT guidelines pointed out that “antipsychotic medications, other than clozapine and olanzapine, are recommended as first-line treatment for persons with schizophrenia experiencing their first acute positive symptom episode” (Buchanan et al., 2010), arguing that both drugs can cause serious metabolic side effects. This recommendation was poorly followed in our study, particularly in the case of olanzapine;: it was included in the 27.1% of the prescriptions and was the most used antipsychotic in monotherapy (n=76). As the PORT guidelines were published in the middle of the inclusion period (January, 2010) and, to our knowledge, this recommendation has not been included in other treatment guidelines to date, this question deserves a special attention in the future studies with FEP.

Regarding the choice of an FGA vs SGA, a recent meta-analysis indicated that, in first episodes of schizophrenia, determinate SGA show superior efficacy, greater treatment persistence and less EPS than FGA, but also a major weight increase and metabolic changes, specially in the case of olanzapine (Zhang et al., 2013). In a multiple-treatments meta-analysis including 15 antipsychotic drugs in schizophrenia, Leucht et al. (2013) also remarked that antipsychotics differed substantially in side effects and that small but robust differences were seen in efficacy, pointing out that classification of antipsychotics into first-generation and second-generation groupings is too simple and uninformative and that the SGA group is too heterogeneous. In any case, from our naturalistic study, it becomes clear that Spanish psychiatrists clearly prefer starting treatment with a SGA, generally in low to medium doses, and that in case of a bad response or side effects, the second option to switch uses to be a SGA too.

Another point of focus in our study is the use of antipsychotic polytherapy. This is a common practice in everyday clinical practice, although there is little scientific evidence to support its use over other strategies (Bernardo et al., 2012; Tani et al., 2013). In our study, this practice was related more to inadequate dosage. Antipsychotic polytherapy was also related to the presence of ADRs, being a more relevant factor than antipsychotic dosage in CPZ equivalents. This result points to the fact that antipsychotic polytherapy is unsafe not only because the result dosage is higher, but for other factors intrinsic to this practice related to phenomena such as drug interactions or mechanism of actions.

Only 8 patients were receiving clozapine. It was used in monotherapy in all cases in low doses compared with other strategies and generally well-tolerated. To our knowledge, there are only 2 clinical trials that have compared the use of clozapine in FEPs; one vs CPZ and the other vs risperidone (Girgis et al., 2011; Sanz-Fuentenebro et al., 2013), pointing to a slightly greater tolerability and greater time in remission efficacy, specifically in relation to treatment adherence. According to these data, low/medium doses of clozapine in monotherapy might be better tolerated than an antipsychotic polytherapy prescription, although the clozapine group is too small to make adequate statistical comparisons.

Another point of interest was to characterize the use of LAIAs formulations in this broad sample of FEPs. These formulations have traditionally been used at latter stages for those patients with schizophrenia with the most severe symptoms, poorest adherence, most relapses, and generally poorest outcomes (Tiihonen et al., 2011; Stahl, 2014). Some clinical guidelines, including the Spanish one, maintain these recommendations (Grupo de trabajo de la Guía de Práctica Clínica sobre la Esquizofrenia y el Trastorno Psicótico Incipiente. Fòrum de Salut Mental, 2009). However, some authors defend that early-phase patients may have the most to gain from LAIAs at a time when their disorder is most treatable and when avoidance of recurrences and rehospitalizations may lead to the biggest gains in outcome (Tiihonen et al., 2011; Stahl, 2014) and decreasing complications associated with noncompliance, such as substance abuse, violence, legal conflicts, and treatment resistance (Zhornitsky and Stip, 2012; Stahl, 2014). Some clinical guidelines, like the Canadian one, are beginning to include the recommendation to use LAIAs in patients in early stages of the disorder (Malla et al., 2013; Manchanda et al., 2013). Despite these reported advantages, only a small proportion (5.2%) of the patients at baseline of the PEPs project were receiving LAIAs, all with risperidone LAI and only one without oral supplementation. Being a 2-year follow-up study, it is possible that this percentage of use of LAIAs will increase in successive controls. This proportion could also be increased with the recent appearance of new second-generation LAIAs, such as olanzapine, paliperidone, and aripiprazole (Patel et al., 2010).

Looking at the use of other psychotropic drugs, a considerable percentage of patients received anticholinergic drugs (12.2%), a percentage that is consistent with the literature about their use in FEPs (Rybakowski et al., 2014). Males received significantly higher doses of antipsychotics and were prescribed anticholinergic drugs more frequently. The presence of 16.4% of affective psychoses in the sample explains, at least in part, the percentages of antidepressant (12.2%) or mood stabilizer (13.7%) prescription, which were clearly associated with this subgroup of patients. Almost 40% of the patients received benzodiazepines. A relatively recent Cochrane’s review about the use of benzodiazepines in schizophrenia and related disorders showed that there is no convincing evidence to confirm or refute its use (Dold et al., 2012). Evidence suggests that benzodiazepines are effective for very short-term sedation and could be considered for calming acutely agitated patients, generally with adequate acceptability (Dold et al., 2012). It should be noted that, in our sample, the use of other psychotropic drugs was not associated to a major risk of presenting ADRs.

Around 35% of the patients reported ADRs; extra-pyramidal symptoms, sedation, and weight gain were the most common. Diverse changes in antipsychotic dosage and new treatments were introduced because of these adverse events. A detailed description of these ADRs and their association with pharmacogenetic characteristics of the subjects will be published in future reports.

A benefit of our study is that it allows a comparison of these prescribing practices in Spain with other similar samples elsewhere in the world. In this vein, the RAISE study recently described these practices in a comparable sample of 404 first episodes of schizophrenia in 21 states in the USA from July 2010 to July 2012 (Robinson et al., 2015). At first glance, there are similar findings between the RAISE and PEPs studies: (1) the proportion of patients treated with antipsychotics was high (83.4% vs 91%), generally in monotherapy (89% vs 81.1%); (2) the 2 more prescribed antipsychotics were olanzapine and risperidone (representing together 53–56% of the prescriptions), but with different proportions in monotherapy (more frequent with risperidone in the RAISE study); (3) women received significantly lower mean doses of antipsychotics; and (4) the prescription of mood stabilizers was approximately 10%. Given that clinical settings in the US and Europe are different, there are significant differences between both studies in some aspects, such as ethnic diversity (much higher in the RAISE study) or patients’ health coverage (which may explain the lower prescription of FGA in Spain 3.7% vs 11.9%, more associated with patients without medical coverage in the RAISE study). A major proportion of FGA prescriptions could also explain that the proportion of patients receiving anticholinergic drugs was higher in the US than in Spain (21.1% vs 12.2%). The use of LAIAS frequencies (9.5% vs 5.2%) could also be different, because paliperidone palmitate was not yet available in Spain during the study period. Other relevant differences may be related to the study design. The presence of an affective disorder (bipolar or unipolar) was an exclusion criterion in the RAISE study, while it represented 16.4% of the PEPs sample. This could explain why there is a greater percentage of olanzapine treatment. Besides different prescription habits between psychiatrists, all patients in the RAISE study were recruited in community settings, which could explain, at least in part, a much higher use of antidepressants (31.9% vs 12.2%) and much lower use of anxiolytic/sedative agents (15.4% vs 38.8%).

There are some limitations in this study that should be taken into consideration. Due to its naturalistic design and by the very nature of the sample included, some recommendations included in clinical practice guideline (such as the convenience of a period of 24–48 hours of observation before starting the antipsychotics, the 12-month minimum duration of an effective treatment, or interventions for detecting and treating low adherence) could not be evaluated in this study (American Psychiatric Association, 2004; Deutsche Gesellschaft für Psychiatrie Psychotherapie und Nervenheilkunde, 2006; Grupo de trabajo de la Guía de Práctica Clínica sobre la Esquizofrenia y el Trastorno Psicótico Incipiente. Fòrum de Salut Mental, 2009; National Institute of Clinical Excellence, 2009). It should also be taken into consideration when extrapolating results that the major part of the participants are tertiary care centers linked to universities and to CIBERSAM, so both the patient sample and therapeutic strategies may differ from those used in other areas.

A strength of our study is that that the diagnostic evaluation was performed with a very comprehensive protocol with strict inclusion-exclusion criteria (Bernardo et al., 2013; Bioque et al., 2013), making this sample much closer to the “real life” FEP population. Because of the heterogeneity of schizophrenia as a clinical entity, the FEP subgroup is of great interest, because it avoids the effect of confounding variables, such as prolonged antipsychotic treatment or chronicity (Bertani et al., 2011; Kapur et al., 2012; Bernardo et al., 2013; Bernardo and Bioque, 2014). Another key feature of this study is that the age of inclusion is wider than in other previous works, including adolescents. To our knowledge, there are no previous, similar studies in Spain. While we cannot claim the sample is representative, all/most participating hospitals cover a health area in the National Health System, and therefore the study may give a good perspective of what is presently the clinical practice in this country.

In conclusion, these results indicate that overall pharmacologic prescription for treating a FEP in Spain follows the recommendations of major medical guidelines for clinical practice. There is a considerable use of anticholinergic and antidepressant drugs, while the prescription of benzodiazepines remarkably high. While almost one-quarter of the patients were treated with antipsychotic polytherapy, only a small proportion of patients was treated with clozapine or LAIAs. More than one-third of the FEP report at least one ADR, being more related to antipsychotic polytherapy patterns than to high antipsychotic dosage.

Together with previous findings, this study supports the future research of determinate pharmacological strategies for the treatment of patients in early phases of psychotic disorders, such as the role of clozapine, LAIAs, and antipsychotic combination, together with security issues.

## Statement of Interest

A. Albacete has received a trainee research staff grant awarded by the University of Barcelona (APIF-UB grants). A. Bulbena has received grants from the Spanish Ministry of Science and Innovation (FIS). A. Gonzalez-Pinto has received grants and served as consultant, advisor or CME speaker for the following entities: Almirall, AstraZeneca, Bristol-Myers Squibb, Cephalon, Eli Lilly, Glaxo-Smith-Kline, Janssen-Cilag, Jazz, Johnson & Johnson, Lundbeck, Merck, Otsuka, Pfizer, Sanofi-Aventis, Servier, Shering-Plough, Solvay, the Spanish Ministry of Science and Innovation (CIBERSAM), the Ministry of Science (Carlos III Institute), the Basque Government, the Stanley Medical Research Institute, and Wyeth. A. Ibáñez has served as speaker or advisor for the following companies: Adamed, Bristol-Myers Squibb, Ferrer, Janssen-Cilag, Lundbeck, Pfizer, Servier, and Otsuka. A. Lobo had a consultancy with Janssen, and he has received honorarium or travel support from Eli Lilly and Bial. A. Mané has received financial support to attend meetings, travel support, and served as a speaker from Otsuka and Janssen Cilag. C.M. Díaz-Caneja has previously held a Río Hortega grant, Instituto de Salud Carlos III, Spanish Ministry of Economy and Competitiveness, and a grant from Fundación Alicia Koplowitz. E. Vieta has received grants and served as consultant, advisor, or CME speaker for the following entities: AstraZeneca, Bristol-Myers Squibb, Eli Lilly, Ferrer, Forest Research Institute, Gedeon Richter, Glaxo-Smith-Kline, Janssen, Lundbeck, Otsuka, Pfizer, Roche, Sanofi-Aventis, Servier, Shire, Solvay, Sunovion, Takeda, Teva, the Spanish Ministry of Science and Innovation (CIBERSAM), the Seventh European Framework Programme (ENBREC), and the Stanley Medical Research Institute. E.J.A. has received grants from the Spanish Ministry of Science and Innovation (CIBERSAM; FIS), the Seventh European Framework Programme, and the Marató TV3. F.C. has received financial support to attend meetings, travel support, and served as advisor or speaker for the following entities: Lilly, Janssen-Cilag, Lundbeck, Otsuka, the Spanish Ministry of Science and Innovation (CIBERSAM) and the Ministry of Science (Carlos III Institute). I. Baeza has received honoraria from Otsuka and Janssen and grants from CIBERSAM, Marató TV3 Foundation, the Seventh European Framework Programme, Fundación Alicia Koplowitz, and Ministerio de Ciencia e Innovación. I. Corripio has received research grants and served as consultant, advisor, or speaker for the companies Otsuka and Ferrer. J. Castro-Fornieles has received grants from the Spanish Ministry of Science and Innovation (CIBERSAM; FIS), the Seventh European Framework Programme, the Marató TV3 Foundation, and the Alicia Koplowitz Foundation. J. Saiz-Ruiz has been a speaker for and on the advisory boards of Lilly, GlaxoSmithKline, Lundbeck, Janssen, Servier, and Pfizer and has received grant/honoraria from Lilly and Astra-Zeneca. J. Bobes has received research grants and served as consultant, advisor or speaker for the companies: AB-Biotics, Adamed, Almirall, AstraZeneca, Bristol-Myers Squibb, Ferrer, Glaxo- Smith-Kline, Hoffman La Roche, Janssen-Cilag, Lilly, Lundbeck, Merck, Novartis, Organon, Otsuka, Pfizer, Pierre-Fabre, Sanofi-Aventis, Servier, Shering-Plough, and Shire, research funding from the Spanish Ministry of Economy and Competiveness –Centro de Investigación Biomedica en Red area de Salud Mental (CIBERSAM) and Instituto de Salud Carlos III-, Spanish Ministry of Health, Social Services and Equality-Plan Nacional sobre Drogas, and the 7th Framework Program of the European Union. M. Bernardo has been a consultant for, received grant/research support and honoraria from, and been on the speakers/advisory board of ABBiotics, Adamed, Almirall, AMGEN, Eli Lilly, Ferrer, Forum Pharmaceuticals, Gedeon, Hersill, Janssen-Cilag, Lundbeck, Otsuka, Pfizer, Roche, and Servier and has obtained research funding from the Spanish Ministry of Economy and Competiveness, Centro de Investigación Biomédica en Red de Salud Mental (CIBERSAM), the Government of Catalonia, Institut d’Investigacions Biomèdiques August Pi i Sunyer (IDIBAPS), NARSAD, and the 7th Framework Program of the European Union. M. Bioque has been a consultant for, received grant/research support and honoraria from, and been on the speakers/advisory board of Adamed, Ferrer, Janssen-Cilag, Lundbeck, Otsuka, and Pfizer. M. Parellada has received research grants and served as consultant, advisor or speaker for the companies: Janssen-Cilag and Otsuka and has received research funding from Instituto de Salud Carlos III, Spanish Ministry of Health (now Spanish Ministry of Economy and Competitiveness), CIBERSAM, Fundación Alicia Koplowitz, Fundación Mutua Madrileña, and has served as a consultant for the EMA. M.J. Cuesta has received research funding from the Spanish Ministry of Science and Innovation (Carlos III Health Insititute), the Government of Navarre and the “Plan Nacional sobre Drogas” from the Spanish Ministry of Health. Maria Paz Garcia-Portilla has been a consultant to and/or has received honoraria/grants from Alianza Otsuka-Lundbeck, CIBERSAM, European Comission, Instituto de Salud Carlos III, Janssen-Cilag, Lilly, Lundbeck, Otsuka, Pfizer, Servier, Roche, and Rovi. P. Ruiz has received financial support to attend meetings by Lilly, Janssen, Shire, and Rovi. A. Alonso-Solís, A. Butjosa, A. Lafuente, A. Llerena, A. Zabala Rabadán, A.M. Sánchez-Torres, B. Cabrera, C. Tapia-Casellas, C. Torrent, E. García Bernardo, E. Grasa, F. Modrego, G. Mezquida, I. González, I. Morales-Muñoz, M. Hernández, M.J. Escarti, P. Gassó, R. Landin-Romero, R. Rodriguez-Jimenez, R. Segarra Echevarría, S. Amoretti, S. Mas, S. Sarró, and V. Balanzá-Martínez declare no conflict of interests.
